# Mesenteric Inflammatory Myofibroblastic Tumor in an Infant: A Case Report and Literature Review

**DOI:** 10.7759/cureus.83700

**Published:** 2025-05-08

**Authors:** Rola Abu Alwafa, Amna Akkawi, Qutaiba Mahmoud, Jehad I Zuhd, Samer Bustame

**Affiliations:** 1 Department of General Surgery, An-Najah National University Hospital, Nablus, PSE; 2 Department of Medicine, College of Medicine and Health Sciences, An-Najah National University, Nablus, PSE; 3 Department of Radiology, An-Najah National University Hospital, Nablus, PSE; 4 Department of Anesthesia, An-Najah National University Hospital, Nablus, PSE

**Keywords:** inflammatory myofibroblastic tumor (imt), mesenchymal neoplasm, multidisciplinary management, pediatric oncology, radical surgery

## Abstract

Inflammatory myofibroblastic tumor (IMT) is a rare mesenchymal neoplasm predominantly affecting children and young adults. Here, we present a case of IMT incidentally discovered in the mesentery of a six-month-old infant, managed through radical surgery. The patient initially presented with decreased oral intake and post-feeding vomiting, leading to the discovery of a large pelvic mass. Following comprehensive evaluation and multidisciplinary management, including surgical excision, the patient experienced a favorable clinical outcome. Histopathological examination confirmed the diagnosis of IMT, highlighting the importance of accurate diagnosis and appropriate management strategies for this rare entity.

## Introduction

The inflammatory myofibroblastic tumor (IMT), a mesenchymal neoplasm, is recognized as a true neoplastic condition by the World Health Organization, falling under the category of neoplastic fibroblastic/myofibroblastic tumors with intermediate biological potential [1,2]. It primarily affects the visceral soft tissue in children and carries a risk of local recurrence, with distant metastasis being rare [1,3]. IMTs have been reported in different body organs, such as the esophagus [4], colon [5], lung [1], uterus [6], and other body sites [2,7,8]. It predominantly affects children and young adults [8], and infantile cases have been reported [9]. We herein report a case of IMT discovered incidentally in the mesentery of a six-month-old infant, managed by radical surgery.

## Case presentation

A six-month-old male presented at a pediatric outpatient clinic (OPC) with a two-week history of decreased oral intake of pureed and mashed food, accompanied by post-feeding vomiting. However, he exhibited good tolerance to breastfeeding. The mother reported no changes in the child's activity levels, bowel habits, or abnormal movements.

Upon evaluation at the OPC, physical examination revealed a guarding abdomen, which precluded deep palpation for masses. An ultrasound revealed a large midline pelvic mass with hypoechoic homogeneous features, regular borders, and internal vascularity, measuring approximately 7 × 3.4 × 5 cm, causing a mass effect on the surrounding bowels. Further investigations, including laboratory tests, indicated an elevated erythrocyte sedimentation rate (ESR) of about 130 mm/h, a high platelet count of 770 k/μL, and alpha-fetoprotein levels of 150 ng/mL.

**Figure 1:**
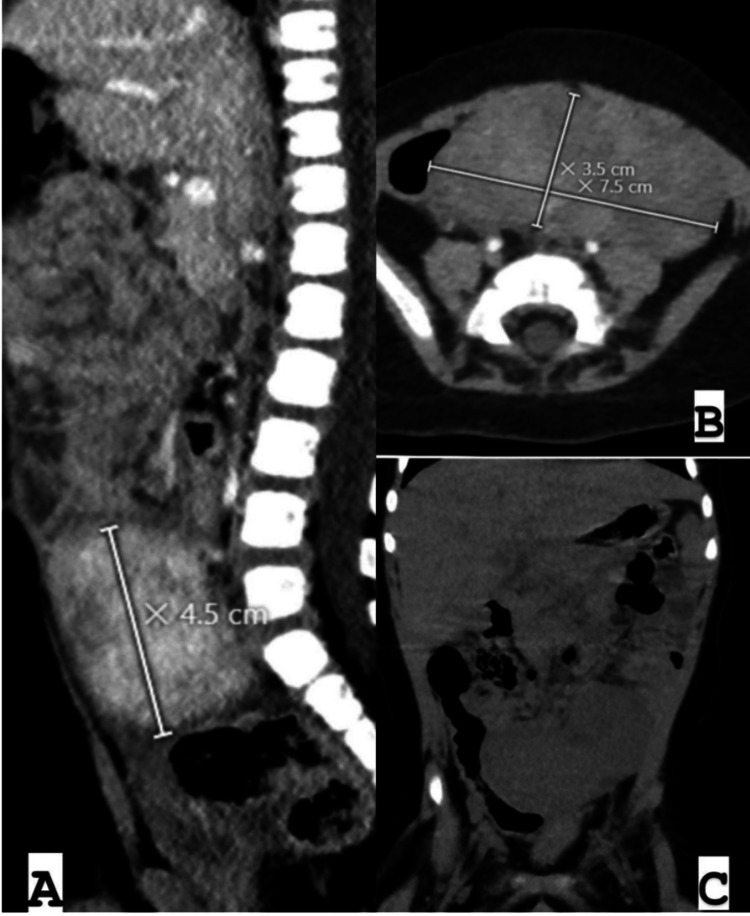
Abdominal CT scan CT scan showed a well-defined, bilobed, heterogeneously enhancing mass lesion measuring approximately 7.5 × 3.5 × 4.5 cm, with surrounding inflammatory changes and free fluid in the left paramidline lower abdomen, anterior to the iliac vessels, displacing adjacent bowel loops (A: sagittal view, B: axial view, C: coronal view).

Subsequent ultrasound scans yielded similar results, leading to an abdominal CT scan, which confirmed a large pelvic mass measuring 7.5 × 3.5 × 4.5 cm, consistent with a neurogenic tumor (Figure 1). The abdominal CT scan demonstrated a well-defined, bilobed, heterogeneously enhancing mass lesion with surrounding inflammatory changes and free fluid. The location of the mass in proximity to the paramidline lower abdomen, anterior to the iliac vessels, and its displacement of adjacent bowel loops raised suspicion for a neurogenic tumor. These characteristics, including the heterogeneous enhancement and lack of significant calcifications, aligned with features commonly associated with neurogenic tumors in pediatric patients.

Physical examination revealed a 9.8 kg infant with no signs of dehydration. Abdominal examination was limited by guarding, which likely obscured the detection of the mass during the initial clinical evaluation. A multidisciplinary team (MDT) comprising pediatric oncology and surgery decided on surgical intervention. On the following hospital day, the patient underwent laparotomy, mass excision, small bowel resection, and appendectomy.

**Figure 2:**
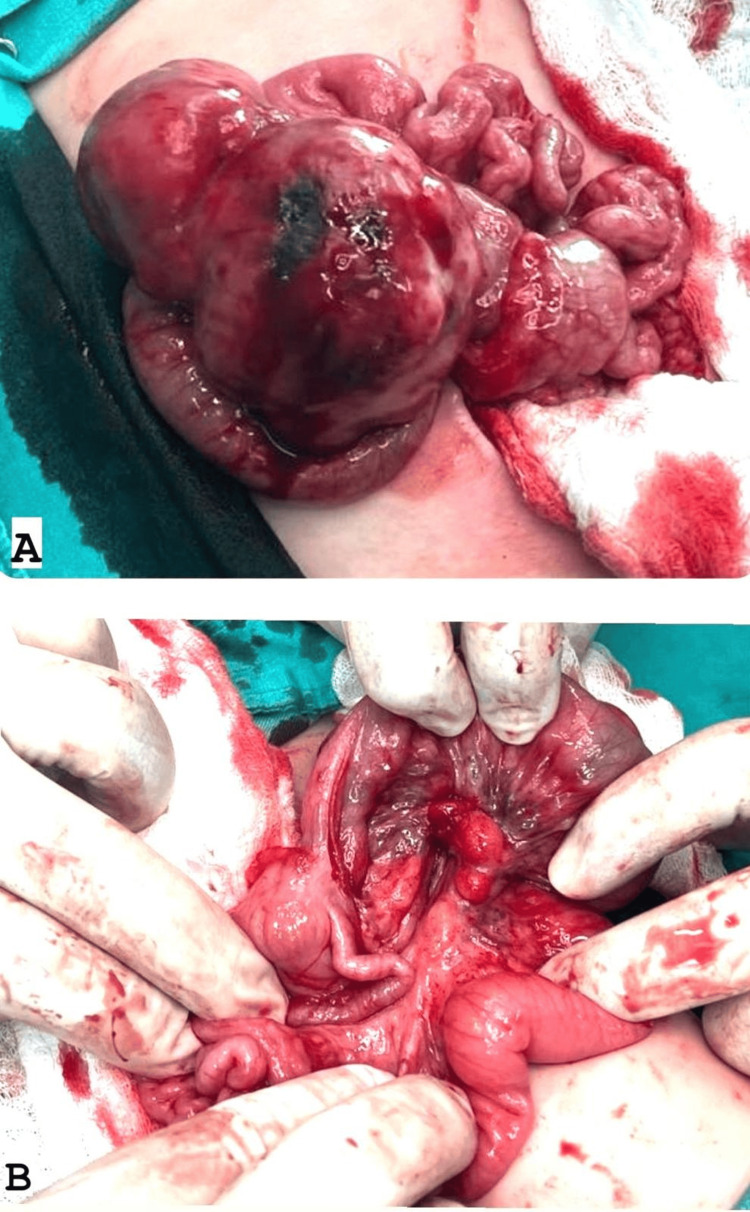
Intraoperative images A: Anterior surface of mesentery. B: Posterior surface of mesentery.

During surgery, the laparotomy revealed an encapsulated mass measuring approximately 7.5 × 3.5 × 4.5 cm, located in the mid-abdomen and originating from the mesentery about 10 cm proximal to the terminal ileum (Figure 2). The mass extended along the mesentery but was distinct from the bowel lumen and displayed a firm to hard consistency. It was successfully enucleated from the mesentery while preserving adjacent structures. The terminal ileum appeared dusky due to compression of mesenteric vessels by the tumor, necessitating a 10 cm resection of the affected bowel segment with primary anastomosis. The appendix exhibited inflammation at its mid and distal tip, justifying an appendectomy. No significant ascites was observed. Multiple enlarged mesenteric lymph nodes surrounding the tumor were noted and biopsied.

**Figure 3:**
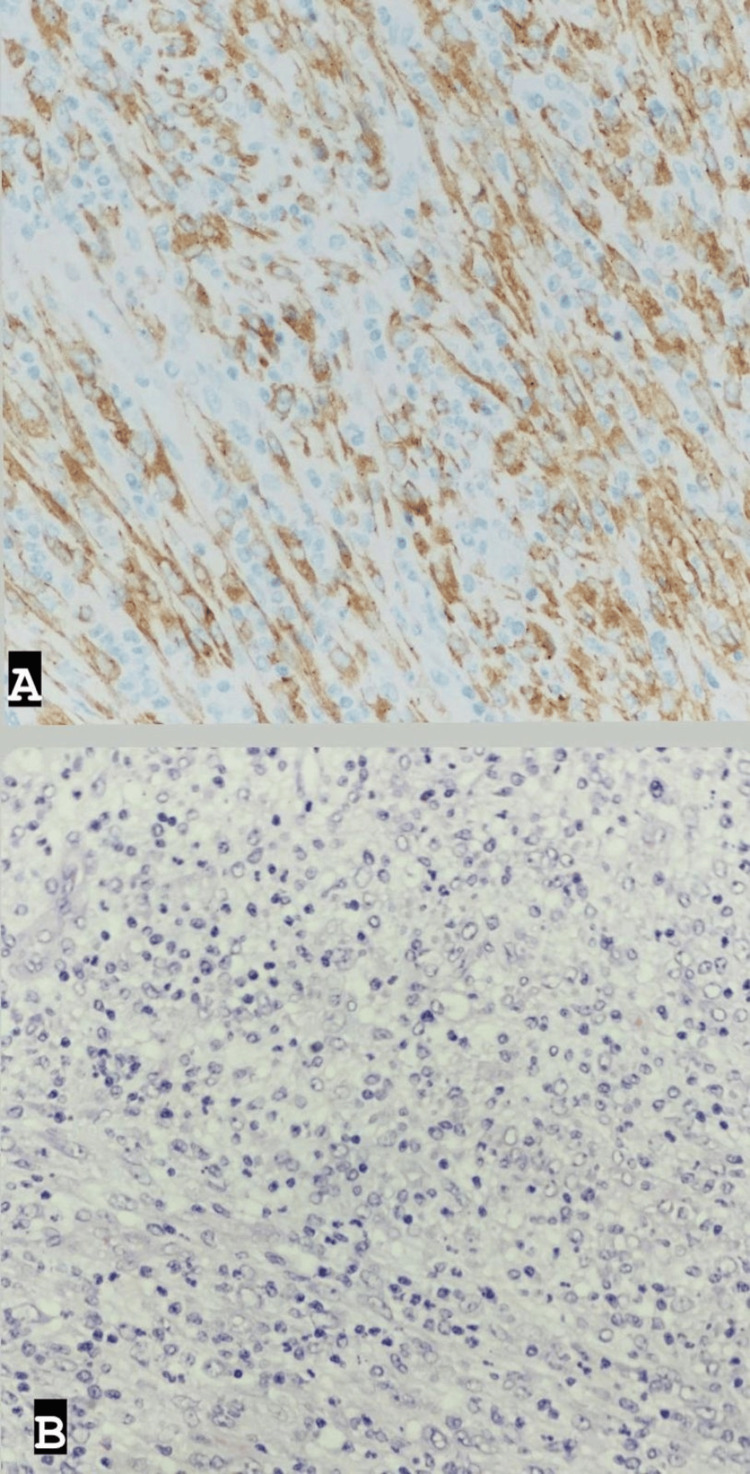
Histopathological sections A: Immunohistochemical (IHC) staining shows diffuse expression of ALK in tumor cells. B: H&E stain shows proliferation of spindled fibroblasts and myofibroblasts in a background of highly vascularized stroma and mixed inflammatory cells.

Post-surgery, the patient was admitted to the intensive care unit (ICU) for close monitoring before being transferred to the ward and discharged in stable clinical condition five days later. Histopathological examination of the excised mass revealed features consistent with an inflammatory myofibroblastic tumor, characterized by a well-demarcated cellular mass with a myxoid and highly vascularized background; spindled fibroblasts/myofibroblasts; and numerous lymphocytes, histiocytes, eosinophils, and plasma cells. Ganglion-like cells were also observed, with 5 mitotic figures per 10 high-power fields. No marked atypia or necrosis was noted. The tumor cells showed diffuse positivity for ALK (cytoplasmic), with rare cells positive for SMA (smooth muscle actin), and were negative for CD30, CD34, S100, Pan-CK, Myogenin, and CD117. CD68 highlighted histiocytes in the background, while CD45 highlighted inflammatory cells (Figure 3).

## Discussion

IMTs are rare neoplasms that were previously classified as inflammatory pseudotumors and are characterized by spindle cells [8]. Children, adolescents, and young adults are the typical age groups affected [8], but several studies have reported occurrences in infants and older individuals [1-9]. IMT can develop in different body locations, with two-thirds of cases involving the abdomen, retroperitoneum, and pelvis [8]. 

The clinical presentation of IMT varies depending on its primary site [3], with fever, weight loss, and malaise being presenting features in up to 30% of cases [2]. Infantile forms of IMT have been consistently reported in the literature [9]. Thrombocytosis, elevated ESR, and polyclonal hypergammaglobulinemia, as observed in our case, are frequently associated with IMT [3]. These findings support the diagnostic relevance of these parameters in identifying IMT.

The diagnosis of this entity depends on proper evaluation using imaging, including computed tomography (CT) and magnetic resonance imaging (MRI) [2,3]. However, imaging alone cannot differentiate IMT from other inflammatory pseudotumors and requires tissue analysis through histopathological examination for definitive diagnosis [3]. This aligns with our case, where ultrasound and CT scans revealed a large pelvic mass, prompting surgical excision for histopathological confirmation.

IMT is considered a distinct pathological entity. Both pulmonary and extrapulmonary IMTs are characterized by rearrangements involving the ALK (anaplastic lymphoma kinase) gene in up to 50% of cases, leading to activation of tyrosine kinase [2]. The typical immunohistochemical profile shows vimentin positivity, focal SMA positivity, and negativity for CD117 and CD34 [3,5]. These findings were consistent with the profile observed in our case, further supporting the diagnosis of IMT.

Mesenteric masses present diagnostic challenges, with potential benign causes such as desmoid tumors and gastrointestinal stromal tumors, alongside malignancies like lymphoma and soft-tissue sarcomas [3]. Inflammatory pseudotumors can mimic peritoneal carcinomatosis, necessitating laparoscopic or open surgical sampling for diagnosis [10]. This was the case in our patient, where surgical intervention was required for both tissue sampling and mass excision.

Complete surgical resection is the preferred curative treatment. Adjuvant therapies such as corticosteroids and chemotherapy may be considered in cases of incomplete resection, with recurrence rates ranging from 15% to 37% within a year [11], largely due to residual disease [2]. Additional therapies, including non-steroidal anti-inflammatory drugs and chemotherapy, may be considered for unresectable or recurrent disease. In our case, complete resection was achieved, and the absence of postoperative complications suggests a favorable outcome.

It is essential to recognize that long-term follow-up is required due to the risk of recurrence. Current recommendations include clinical examination every three to six months and follow-up CT scans at six-month intervals.

## Conclusions

Our case underscores the diagnostic and therapeutic challenges associated with IMT. With prompt recognition, multidisciplinary collaboration, and surgical intervention, favorable outcomes can be achieved. This highlights the importance of comprehensive evaluation and tailored management strategies to ensure optimal patient care.
